# Dr. Beall’s Trip to Europe

**Published:** 1890-10

**Authors:** E. J. Beall

**Affiliations:** Liverpool, England


### Correspondence.

### DR. BEALL'S TRIP TO EUROPE.

LIVERPOOL, ENGLAND, August 30, 1890.

*Editor Daniel's Texas Medical Journal:*

My four months sojourn across the Atlantic nears the end. Men and methods relating to medicine are graven upon the tablet of memory; these I hope in the coming days to recall as satisfactory pleasantries, as gratification to a mind so long engaged in the same line of work as those with whom I have moved in the past just behind me.

In writing you a second letter, in which I will refer to the International Medical Congress at Berlin, and other matters of professional interest, I do not imply any reflection upon you or your spicy journal; for you and they know full well that the work of that great and important body is yet measurably in a foreign language, and still in the hands of the Secretary of the Congress being prepared for publication; that it is only through correspondents who participated in the meeting, that any of the proceedings is known to the profession who were not present.

Arriving at Berlin from Dresden, early Sunday morning, August 3rd, in company with Dr. Ryburn, of Illinois, and ladies, we took carriages and rode rapidly over the great and thriving metropolis of the German empire. Its public buildings, magnificent gardens and parks teeming with the population of Berlin, enjoying the pastime and music of the weekly holiday, was interesting to behold. This gave us an insight to a custom, which rivets the Teutonic race to the "Fatherland." A custom of their early home-life to which they recur in mind and practice all through life, no matter under what sun or flag they may live.

The Congress was opened in the Circus Renz, the day after our arrival. In Karlstrasse, after turning to the left out of Friedrichstrasse, we find a deep opening leading to the circus. At the beginning of this opening, there was erected a triumphal arch, from which beautiful garlands were hung to the very entrance of the building. Just to the side of the opening to the Congress, was Lasser's private hospital, masked in garlands of flowers of every hue and odor, and there could be seen in the midst of this æsthetic display in golden letters, the welcome: "*Orbis Terrarum Universi Medici, Salutamini.*"

Within the courts, at the foot of the steps of the various aisles leading to the circus, were tables laden with salads and viands of many flavors and kinds. Here were also placed well chosen ornamentations, and social groups gathered in this large crescent court just outside the great room, within which met the greatest body of scientists of which ancient or modern times gives us any record. The decorations, flags of all nations pendant from the high ceilings, emblems, etc., within the circus were appropriate, beautiful, magnificent. In the background the "Baths of Caracalla" were painted. In front, overlooking the platform, was a large figure of Æsculapius, and upon his trident there glistened the emblem which adorned the coat lapels of the members. In the royal box, on the left, decorated with superb plants and flowers, was the bust of the young emperor; the opposite box, resembling a temple, showed Minerva in the foreground. The sides of both boxes were embellished with bas-reliefs representing German victories. Within these walls thousands gathered, among whom several hundred ladies were sprinkled. In the arena reserved for the civic and military dignitaries, there could be seen many whose names honor medicine and surgery—honor themselves, and the nations which claim such giants in intellect, and such workers, directly and indirectly for the brotherhood of man. In this great galaxy you could see Billroth, Meynert, Nothnagel, Bardaleben, Kuester, Olshausen, Esmarch, Kraft-Ebing, of Austria; Lister, Paget, Horsley, Simon, Pavy, McCormack, Clark, McEwen, of England; Dujardin-Beaumetz, Bouchard, Apostoli, Ollier, Roux, Cornil, of France; Baccelli, Cantani, Maragliano, Golgi, Foa, Celli, et al., from Italy; Van Beneden, Thiry, of Belgium; Rosenstein, Snellen, Stockvis, Pel, Forster, Pekelharing, of Holland; Iversen, Gent, Lange, of Denmark; Klaus-Hassen, Heiberg, Laache, of Norway; Ketzius, Holmgren, Axel, Key, of Sweden; Krocher, Fehling, Sociux, Cordes, DeFour, of Switzerland; Schmidt, Barfut, Danilewski, Puritz, et al., from Russia; Knapp, Senn, Keen, Pancoast, Jacobi, Loomis, O'Dyer, Osler, Tyson, Engelmann, et al., from America; Canada, Mexico, Spain, Turkey, China, South America, Japan, in fact, every civilized nation in Christendom was represented.

Long before the arrival of President Virchow and Secretary General Lasser, the house was filled from arena to uppermost gallery. The diplomatic corps was conspicuous. The German government was represented by Minister von Boetticher, and State Secretaries Maltzan and Oehlschlaeger; the Prussian government by Heerfurth, Miquel and Gossler; the army by Generals Rauch and Pape; the city of Berlin by Burgomaster Forckenbeck, and many others. The great German universities had their rectors and deans in official robes. The Duke Karl Theodor and President Virchow entered the building together and were loudly greeted by the multitude; and when the venerable pathologist, anthropologist and statesman ascended the platform, the greeting was indeed deafening.

I wish I knew the language in which is embodied so much science, sociality and humanitarianism as in that of the German, that I could give you a correct and faithful epitome or full translation of the grand address of the great Virchow on the occasion of the opening of the Tenth International Medical Congress. This task, or pleasure, linguistic knowledge debars me from. I fear, those of us unacquainted with the German language, when forced to rely upon translations for the sentiment, depth of thought, comprehensiveness and adaptability to the occasion and times will lose much of the address of grand old Virchow.

His welcome to the Congress, his reference to those whom he had helped to educate while a teacher more than forty years in a German university, when he spoke of medicine as being one of the sincerest representatives of humanity, when he referred to the fact that medicine was a popular science in Germany, that it was the first International Congress which had even in its inception the sympathy and material aid of government, these together with many other thoughts came forth in words that burned and thrilled, and as a master-piece of thought will live in print and in minds when jealousies, ingratitude and ordinary efforts are fading and lost in the vanishing past. His transcendently powerful effort closed with this admirable sentiment:

"Having referred to the political peace of Europe * * * we are happy to see ourselves surrounded by so great a number of valued professional brethren, in whom we are permitted to presuppose there exists a desire for peace, for peace nourishes and dissension destroys, who desire to live in accord with all the world in order to pursue the tasks of science and the aims of humanity undisturbed and in our own way, a co-operation to this end will prove a stimulus to diligence—once more therefore, I bid you hearty welcome to our city! May each day contribute more and more to promote full understanding and true friendship amongst us all!"

Dr. Lasser, Secretary General, then delivered his report on the internal affairs of the Congress.

Secretary of State von Boetticher welcomed the assembly in the name of the German empire, by order of the emperor, who regretted being unable to attend in person.

Minister von Gossler welcomed the Congress in the name of the Prussian government.

Burgomaster von Forckenbeck welcomed for the city of Berlin, and gave a condensed account of the hygienic institutions of the city.

Dr. Goff welcomed the Congress in the name of the German societies of physicians and surgeons.

Dr. Hamilton of the "Ninth Congress" then spoke. He was followed by Sir James Paget, the veteran English pathologist, who ascended the platform amidst loud applause, and expressed the thanks and guaranteed the co-operation and sympathy of the English profession. Dr. Bouchard responded for France. Then came the powerful oration in classical Latin of Baccelli for Italy. He is certainly the most impassioned graceful declaimer I have ever heard, excelling Semmola, whom many admired so much at Washington for his graceful declamation and native fire of oratory. Csatary followed for Hungary. Many notable and great men responded for other countries.

After the unanimous election of Prof. Virchow as permanent president, the honorary presidents were elected. The vice-presidents were: Stockvis, Baccelli, Billroth, Bouchard, Billings, Stokes, Paget, Granger, Stewart, Duke Karl Theodor of Bavaria. The general session then adjourned for lunch to the well laden tables in the outer court of the Circus Renz. Then to the Ausstellungs-Park, Inabnit, where the twenty sections were merged into active work.

The exposition building here was well adapted for the numerous sections. Separate halls, ornamented with plants, flowers and fountains, and adorned with beautiful paintings and artistic works in marble.

Surrounding this immense building, within which were minds, busy as bees, solving the problems that intimately concern human life and happiness, merging hypothesis into facts, were beautiful gardens, and all the comforts that the animal part of the human organism could desire. A feast for the mind within, a feast for the body without.

One of the larger halls was set aside as a committee room for the ladies. This hall was beautified by a magnificent central fountain, and adorned with paintings and statuary, which only masters could have produced. This committee, of which Mrs. Leyden was president, was organized for the convenience, comfort and purpose of entertaining the wives and daughters of members of the Congress.

Outside of the vast and beautiful structure, in which convened the sections of the Congiess, were the rooms containing the displays of the appliances of medicine and surgery, including military and hygienic apparatus and conveniences of numerous and varied kinds. Hospitals erected on railroad tracks, field hospitals of convenient construction with the comforts of permanent hospitals, and the latter I saw in the yards of the Augusta hos pital. These were well adapted for septic and special cases and convalescents. Numerous were the displays from many countries, showing new, approved and useful instruments, apparatus, etc.

Since leaving Berlin, I have learned that through the influence of Prof. Virchow and others, the emperor has ordered that the display at the Congress shall remain intact as a nucleus for a museum for all time. This is in keeping with the ideas of the young emperor, who, like Napoleon I., in addition to the milk of human kindness in his nature, would preserve the stability and strength of his nation by conserving health and strength through the medium of every well authenticated means for furtherance of public hygiene and the prophylaxis of disease.

It is well known that a number of addresses were delivered in the general sessions, of which four were held. These addresses, on account of the reputation of their authors, have been translated, and pretty well disseminated throughout Christendom. We are glad to know that our countryman, Dr. H. C. Wood, of Philadelphia, delivered one of the general addresses, and in so doing he forfeited none of his prestige as a therapeutical observer. His subject was anæsthesia. He announced some rather startling views—views that are at variance with the custom of many who use chloroform and ether. He does not admit that respiratory failure always occurs when death takes places during chloroform anæsthesia. In this he differs somewhat from the results of the Hyderabad Commission. He claims that death may ensue sometimes from heart failure, and at other times from respiratory failure. He presented diagrams showing alteratiods in blood pressure during anæsthesia after the inhibition of various remedies, such as alcohol, digitalis, strychnine, morphia, atropia, etc. He deprecates and warns against the use of whiskey and morphia before and during anæsthesia, and claimed that his experimentation showed that strychnia and digitalis were the remedies to be relied upon. How common has been the practice of surgeons since 1847, till this day, to administer whiskey and morphia before using chloroform ! This was the invariable practice of the late Dr. Warren Stone, during the years in which we were familiar with that strong man's work.

I shall refer to only one other paper presented in the general session: that of Dr. Robert Koch. I am influenced to do this from the fact that there seems to exist throughout America a widespread misinterpretation of that scientist's views. This I infer from the letters of inquiry I received upon my return to London, and from conversation with a few medical men who were not at the Congress.

I did not understand that Dr. Koch, in his address, stated that he had *discovered a remedy for tuberculosis*. The deductions from the translations I have read fixes in my mind something like this: That for many years he had been seeking agents for the theraphy of consumption, and in doing so had begun with pure cultures of tubercle bacilli. He had found that tar pigments, mercurial vapor, certain etherial oils, the salts of silver and gold, especially cyanide of gold, prevent the growth of the bacilli, which would therefore be expected to keep the disease in abeyance or at a standstill. When the remedies, so effectual in the culture bottles were used against the bacilli in the bodies of animals, they proved inefficient. He said he continued the research, and had found what he had sought. In experimenting with animals, especially the susceptible guinea pig, he could not successfully inoculate that or other animals when they were treated with the substances in question. That even when the disease was far advanced it could be brought to a standstill with the treatment. This fact he hopes will give rise to search for effective remedies in other infectious diseases; and claimed for such work a field for an international contest of the highest and noblest kind.

Dr. Koch *did not divulge the remedy* with which he arrested tubercular processes in animals; but indulged the assertion that he had great hope that a cure was possible for this disease. His care and precaution in regard to a therapeutic success in tuberculosis, or in giving publicity to a remedy, in which doubtless he has great hopes, is but in keeping with the custom of this great scientist. We well know that the discovery of the tubercular bacillus, and also that of the coming bacillus was a secret of his laboratory for several years, and that neither was given to the world until he was satisfied that when promulgated, his views would not be successfully controverted, but instead, corroborated by contemporaries in the same field of work. This we know was done with but few respectable dissensions.

Fearing that your readers, or at least a portion of them, may not have had access to journals, translations, etc., I would like very much, if time and space did not interdict, to review many of the papers of the sections as well as those of the general sessions; but being necessarily limited, the mere reference to them here may stimulate them to an industrious effort to procure the literature that will furnish the brainwork of the representative men who participated with pen and voice at the Congress.

Seven hundred papers were read and discussed. Whilst out of this mass of brainwork it cannot be said that anything startling was presented, yet many hypotheses were strengthened, and peradventure some facts were firmly established.

The papers of Dr. Grainger Stewart, of Edinburgh, M. Bouchard, of Paris,—the former upon "Bright's Disease," the latter upon "The Mechanism of Infection and Immunity"—the address of Mr. Axel Key, of Stockholm, on "The Development of Puberty and its Relation to Morbid Phenomena Among School Children," will deserve careful reading when they can be obtained.

In the gynecological section I heard the paper of Cushing, of Boston, upon "The Drainage-tube in Laparotomies." The paper referred to his personal experience. He claimed good results from the drainage tube; insisted that it should not be too large; and that it should be introduced deep into the pelvis. This, he emphasized. Mr. Tait, in discussing the paper, stated that he was a strong advocate of the tube, particularly in subjects past forty years of age; said he had learned the use of the drainage tube from the elder Keith. Mr. Bantock, my beau ideal of an abdominal surgeon, also advocated the tube. He has a peculiar mode of using it, together with his dressings, which I have seen him apply many times at the Samaritan hospital, and greatly pleased with his method. Other men of reputation responded to Cushing's paper; but they spoke in languages with which I am not sufficiently familiar to attempt a synopsis or even a reference to the pertinent points made. I shall look forward to the transactions for elucidation with much interest.

In the same section Mr. Tait made a verbal report of cases in abdominal surgery. He said that he was an heretic in regard to antiseptic surgery. That he believed in the development of micro-organisms in necrotic tissue, but not in live tissue; that he did not operate upon dead tissue, hence, had no fear of micro-organisms. Some years ago, in my report as Chairman of the

Section on Surgery of the Texas State Medical Association, I discussed Mr. Tait upon this subject or rather referred to his custom or habit in surgery, hence, will not now speak of his stubbornness in the line of asepsis and antisepis. It seemed from the emphasis he placed upon the removal of ovaries and tubes in miomata, that he wished this to be considered the ultimatum of his life work. He stated, that early in life he had begun this operation to accomplish good results in the cases to which he had just referred. That he was aware other means had been brought forward to accomplish the end he sought; but that he preferred to perfect one plan rather than jump about from one measure to another as they bobbed up in the surgical field. He knew good results were reported from Apostoli's method, but he had no personal experience with that treatment.

Dr. A. Martin replied to Mr. Tait. He stated that he and his German colleagues had given Mr. Tait's methods a fair trial in Germany; but had never had the results which Mr. Tait claimed. But as Mr. Tait had come to the Congress and emphasized the results he had formerly reported, he (Martin) and his colleagues would make another attempt at corroboration.

It was evident to my mind that Martin did not believe Tait's statistics; and although he was as polite as practicable in his reply, Mr. Tait being a guest of the Congress at Berlin, Martin's home or city, I could not but think that behind Martin's face and words he did not believe a word of what Tait said. I heard many hard things of Mr. Tait in London, Berlin and elsewhere. I hope they are not true. They did not come under my personal hearing or observation, and I shall not repeat them.

Upon the whole, the Congress was a grand success—both scientifically and socially. The leaders of the profession in Berlin vied with one another in hospitality to their guests, and the Congress was the means of establishing good fellowship among the medical men of the various countries represented. It was a notable and pleasant feature to observe the courtesy and cordiality shown the members of the Congress from France. It will tend greatly to mollify the unpleasant feelings and impressions which grew out of the Franco-German war.

As you already know, the Eleventh International Medical Congress will convene at Rome, the "Eternal City." With Baccelli as president, and with the history of that city, its galleries, sculpture, schools of learning and literature, the doctor who can should by all means go and revel in the sights and the condition that will be presented for his edification at the coming meeting of 1893.

The great energy and industry displayed by the German profession in the scientific work of the Congress was unanimously conceded to be without precedent in the history of its organization. Nor did they lag in the social features of the Congress, which were grand beyond conception. The entertainment at Rathan's on Monday night; the dinners the succeeding nights of the week, ultimating in the final entertainment at Kroll's garden on Saturday, when prima donnas from every country of Europe were imported—even the bonded songstress of Dresden—to entertain us with song; yards lighted to the very heavens with electricity, plants and grass glistening with natural and electrical flowers, tables laden with food and drink—all conspired to render the entertainment a fitting end to the great work of the week.

I lingered at Berlin after the Congress terminated; and the letters tendered me by friends in New York and London gave me unbridled liberty at seeing much of the men and their methods at Berlin.

At the Augusta I was kindly entertained by Dr. Kuster, and saw much of his work. He is one of the best surgeons I have ever met, and is the life of one of the best arranged hospitals I have ever seen. The Augusta is situated in a beautiful park, and while it is not so large as some of the other hospitals in Berlin, its clinics are surprisingly large. Day after day the operative clinic would begin at 1:30 o'clock and never end until nearly or quite sundown. I cannot refrain from referring to some of the views and work of Dr. Kuster. He showed me a number of cases of senile enlargement of the prostate gland which he had entirely relieved by operation. He makes a circular anal cut, extending a linear one to the coccyx and scrotum, dissects down to the prostate and removes conical sections from the lateral lobes. This presupposes that the lateral lobes are principally enlarged, which he says is often the case, and may be determined by rectal examination. If the middle lobe is affected, he reaches and reduces it by means of a supra-pubic cystotomy. His results are all one could wish. In exsecting bowel for malignancy he varies the procedure from what I saw Pean, in Paris, and Allingham, in London, do. He cuts or chisels off the coccyx, and if necessary, by reason of extension of the disease, does not hesitate to remove a portion of the sacrum so that he may thoroughly remove all disease. This I saw him do in one case. He presented me with a monograph relating many, very many such cases. In a case of lingual cancer he excised one-third of the inferior maxilla (doing a preliminary laryngotomy) and removed the entire organ. The patient was doing well when I left Berlin.

Dr. Kuster is not a strenuous advocate of operative measures upon children who are subjects of tubercular disease of joints. I saw many cases in which he would introduce five or six drachms of an emulsion of iodoform (iodoform 30, glycerine 130) by means of a hollow needle attached to a large glass hypodermic syringe. He repeats the procedure at intervals of four or five weeks. For the hip-joint he selects a point at the inner border of the sartorius muscle, high up, just below Poupart's ligament. At the knee-joint, if pus is present, he introduces the needle, withdraws the pus with the syringe, detaching barrel from the needle without removing the latter, and when the joint is evacuated throws in the emulsion. He claims good results. Mr. Barker, of University Hospital, London, merely cuts down to the joint, curettes and irrigates, removing necrotic tissue and loose bone, throws in the emulsion of iodoform and closes with suture.

I have heretofore referred to Barker's irrigating spoon. As I neglected to secure one while in London, I have taken steps to have a slightly modified one made by Aloe, of St. Louis. I consider the instrument one of the best new things I saw during my absence, and earnestly recommend it to the profession for many purposes.

I saw two cases of actinomycosis in the human subject—one at Israel's clinic, and one at that of Kuster. I also saw mountings of stained specimens of the micro-organisms in both cases. In the case Dr. Kuster treated he used the knife freely at two points of the abdomen and a point in the femoral region, curetted thoroughly and used Paquelin cautery. The case looked very unpromising.

Hahn at the Friederich-Hohe, is the spirit in surgery of that extensive cottage hospital. I witnessed many important operations by him; and he was very kind and courteous to American callers. Prof. Prewitt, of St. Louis, and I had a very pleasant time at this and the Augusta hospital. We saw a case of practical importance and such as often occurs to the railroad surgeon, pass through the hand of Hahn. A man was brought in who had sustained a railroad triple injury. The left arm was crushed at the shoulder; the left leg crushed at the middle of the leg, and right foot crushed. Instead of making immediate amputations he cut off the hanging members with scissors, ligated the important vessels liable to bleed when reaction should come, dressed antiseptically, bandaged, had patient wrapped warm, put to bed with external heat applied, and awaited the occurrence of a condition in which he could tolerate proper amputation. I was pleased with his management of the case, and suggest it for adoption. However, this case survived only five or six hours from the time of accident.

An interesting case was presented in the person of a woman whose left breast and axillary glands had been removed eight months previously by Hahn, for cancer. She now returned with same disease in right axillary glands, but not in right breast. He removed the breast and glands both. Was this a primary infection? or was it secondary to the disease for which the first operation had been done? Dr. Hahn emphatically sustained the latter hypothesis!

I visited Von Bergmann's clinic a number of times, and there noticed an innovation from most recent operations (although at one time in vogue with many); i. e., stuffing the wounds of operations with antiseptic gauze and leaving them so for several hours before applying the permanent dressings.

I was very much impressed with the great size of the tracheotomy tubes used by the German surgeons. This was observable at both the Augusta and Friederick's-Hohe hospitals. Their surgeons wrap the instrument with antiseptic gauze or lint before forcing throufih the cut in the trachea. The tubes I saw used several times in London are larger than those we are accustomed to in America, but not so large as the ones used in Germany. I saw Pean, of Paris, insert a very large tube in a case of tnbercular disease of the larynx.

During the second Sunday of my stay in Berlin, I visited Dr. Israel's clinic; and in my whole life have rarely seen such an interesting collection of cases. They were cases brought together for the purpose of observing results after weeks, months and years had elapsed since the date of operation. No operation was done during the clinic, but case after case was brought in that we might see the result of work at a time subsequent to the date of operation. We saw many cases in which he had removed a kidney; a great many in which he had performed operation upon the hip and knee joints; a number in which he had done plastic operations for restoration, or for forming noses. We were pleased with the results; and I heard a St. Louis surgeon remark that in his mind they stamped Israel as a *natural* surgeon. Dr. Israel showed me the entire removal of the facial nerve with branches in a manner that was new and novel to me. An incision was made below the malar bone, the nerve seized by a slender parallel bladed pair of forceps, then by turning the forceps laterally over and over the nerve and all its branches external to the infra-orbital foramen were completely wrapped there around. I saw the nerve thus removed afterward spread upon a plate and covered with gelatine; and it was indeed a splendid specimen for the anatomical study of that nerve.

All persons upon first visiting the Olshausen clinic, as soon as they are registered, are presented with a card which states: It is expected that visitors shall have had a bath, changed their linen; that they shall not have been about infectious diseases, nor in a dead-room within the two days immediately preceding their visit, and that they shall remove coat, vest, suspenders and cravat before entering the operating room. This card was written by Schroeder. Upon my first visit to the operations of Olshausen, I was presented with one of these cards which at once reminded me that Mr. Bantock, of the Sanitarian, London, requires a similar course to be followed by those who visit his operations. On one occasion, at the Great Ormond St. Hospital for Children, London, while I was giving attention to internal medicine with Dr. Barlow, I found myself almost in the dead-house, at an autopsy in a case of diphtheria. I suddenly recalled that I would be with Mr. Bantock the following day when he would do an abdominal section. I did not enter the room; but the proximity with which I came, caused me to repair to a bath-room and make a complete change of toilet, before going to the Sanitarian. I hope Mr. Bantock will not hear of this acknowledgment; for I very much admire the man and his methods, and would dislike for him to know that I ever jeopardized his statistics by a little absent-mindedness.

The radical cure of hernia by operation which seemed to have taken hold of the surgeons of America several years ago, is found much in vogue in Europe, in all countries, and with many operators. This impetus for radical relief, which in the past was relegated to the truss-maker, for treatment but not cure, can be accounted for in no way other than the safety with which operations can be done by the methods of modern Listerism, compared with the surgery of pre-antiseptic days. The more accomplished operators have some special technique for the radical cure of hernia; but when analyzed, little or at least unimportant variation from the methods known as McEwen's and McBurney's, or what may be called the British and the American methods, will be found.

At Westminster I saw Mr. Davis suspend a child in Sayre's jury-mast for the purpose of dividing the tendons in a case of torticollis. I do not commend the plan; merely mention the fact to indicate an Americanism in London. It protracts the operation, and limits it to a tenotomy that may not be complete. 'Twere better to make an incision, under proper precautions, that the tenotomy may be done surely, under the eye.

We know that Wyeth and others, in America, have had an experience in wiring the patella in cases of its fracture. That their results were such, they do not very highly recommend that procedure in such injuries. But in London it is different. Cases of wiring occurred under my observation at King's College Hospital, in the service of Sir Joseph Lister, as well as that of other metropolitan surgeons.

The great amount of tubercular disease prevailing in Europe, affords one a fine opportunity to see the surgical methods essayed in such cases. In any city one may visit one will find it convenient to witness excision of joints. There will be observed some variations in technique, according to the operation and operator. Hahn, at Berlin, resorts to the plan advised by Wyeth, and so often done in New York, using steel nails through the head of tibia and into the condyles. In London, the plan usually resorted to is to saw through the patella, either transversely or horizontally, remove diseased membranes, cartilages and bones, wire and dress in fixation splints, either plaster of paris, supplemented with iron strips, or a Thomas splint. They are sanguine of success in most cases.

At the Royal Opthalmic hospital, London, I met friend Chilton, of Dallas. This is perhaps the greatest field in the world for this specialty. I very much enjoyed the clinics there; and greatly admired the kindness, politeness and skill of Mr. Teay-Lang and others.

I will refer to one more city, and the clever surgeons whom I there met, it being the city at which, while awaiting the good ship, City of Norfolk, I began this letter.

At Liverpool there is being built one of the model hospitals of the world. A committee was sent through Great Britain and the continent of Europe for the purpose of investigating hospital designs and systems, and the result of their work is the Queen's Hospital at Liverpool, which is now nearly completed. It is certainly a beautiful piece of handiwork, and will contain and furnish facilities possessed by few hospitals.

In the old hospital I saw Mr. Paul resect the colon for obstructive disease, a stenosis of several days standing. He adopted the decalcified bone process, as devised by Senn, of Milwaukee, using both Czerny and Lambert sutures. The operation lasted one hour and a half, and when completed, the patient, a woman, was put to bed in good condition.

I had the pleasure of assisting Mr. Parker, with whom I became very much in love, in a beautiful operation upon a boy, who, two years previously received an injury, which resulted in urethral fistula. There had been great loss of structure from the early urinary infiltration and sloughing. Mr. Parker did me the honor to accept my suggestion of an Americanism (Marcy's buried suture), and when the operation was completed, the parts looked well, and I am satisfied, will result in permanent relief to the lad. I am promised in the near future a letter giving me results in these two cases, the last I witnessed ere I returned to America.

I might extend this letter, written *currente calamo*, and which I know is very disconnected, speaking here and there of work I witnessed abroad; might extend the scribbling into internal medicine, to which I generally devoted the mornings, but I fear you will conclude there is "too much smoke for so little fire," and I know you have a waste-basket hard by, and a gas jet overhead; hence, I will close with this suggestion: When your readers have read all the good articles in your journal, if any time is left for which they have no especial use, they may then go to sleep over or wade through this letter with the hope that some idea may be found; and even though disappointment follow the reading, 'twill not be the first time in life such have wasted the gliding moments.

Yours truly,

E. J. BEALL.

